# Lipase Production by *Limtongozyma siamensis*, a Novel Lipase Producer and Lipid Accumulating Yeast

**DOI:** 10.4014/jmb.2304.04006

**Published:** 2023-07-10

**Authors:** Varunya Sakpuntoon, Savitree Limtong, Nantana Srisuk

**Affiliations:** 1 Department of Microbiology, Faculty of Science, Kasetsart University, Bangkok 10900, Thailand; 2 Biodiversity Center Kasetsart University (BDCKU), Bangkok 10900, Thailand; 3 Academy of Science, The Royal Society of Thailand, Bangkok 10300, Thailand

**Keywords:** Lipase, lipid, low-cost substate, optimization, production, yeast

## Abstract

Lipase is a well-known and highly in-demand enzyme. During the last decade, several lipase optimization studies have been reported. However, production costs have always been a bottleneck for commercial-scale microbial enzyme production. This research aimed to optimize the conditions for lipase production by *Limtongozyma siamensis* DMKU-WBL1-3 via a One-Factor-At-a-Time (OFAT) approach combined with statistical methods while using a low-cost substrate. Results suggest that low-cost substrates can be substituted for all media components. An optimal medium was found, using response surface methodology (RSM) and central composite design (CCD), to consist of 0.50% (w/v) sweet whey, 0.40% (w/v) yeast extract (food grade), and 2.50% (v/v) palm oil with the medium pH adjusted to 4 under shaking flask cultivation. From an economic point of view, this work was successful in reducing production costs while increasing lipase productivity. The medium costs were reduced by 87.5% of the original cost while lipase activity was increased by nearly 6-fold. Moreover, lipase production was further studied in a 2-L stirred-tank fermentor. Its activity was 1,055.6 ± 0.0 U/ml when aeration and agitation rates were adjusted to 1 vvm and 170 rpm, respectively. Interestingly, under this optimal lipase production, the yeast showed accumulated lipids inside the cells. The primary fatty acid is a monounsaturated fatty acid (MUFA) that is typically linked to health benefits. This study hence reveals promising lipase production and lipid accumulation by *L. siamensis* DMKU-WBL1-3 that are worthy of further study.

## Introduction

The genus *Limtongozyma* was first described in 2019 as a new yeast species, *Limtongozyma siamensis*. It was discovered during an investigation of a lipase-producing yeast community in a grease trap, which is a lipid-rich environment [1]. Currently, this genus comprises only two species, *L. siamensis* and *L. cylindracea*, of which the latter is the type species of this genus. The yeast, *L. cylindracea*, was initially classified as a *Candida* species during a search for lipolytic yeast by Yamada and Machida in 1962 and this discovery was later validated by Meyer and Yarrow in 1998. Finally, this yeast was transferred from the genus *Candida* to *Limtongozyma* in 2019 [1]. According to the isolation source and aims of the investigation, it is not surprising that *L. siamensis* and *L. cylindracea* are lipase producers. Extensive research on lipase production by *L. cylindracea* has been reported [2-7] whereas that by *L. siamensis* was primarily reported on a solid medium only during the description of this novel yeast species [1].

Lipases (E.C. 3.1.1.3) are hydrolytic enzymes with potential applications in various industries [8, 9]. The function of lipase is to catalyze the hydrolysis of the ester linkages of long-chain acylglycerols at an oil-water interface. Lipases from various sources showed diverse properties in terms of substrate specificity (fatty acid specificity in esterification reactions), positional specificity (the position of the ester bond hydrolyzed by lipase), optimal pH, and thermostability [10]. The sources of lipases are wide-ranging, including plants, animals, and microorganisms. However, microbial lipases are more useful than those from other sources due to their higher yield, the simplicity of genetic manipulation, the wide range of catalytic activities, and the fact that their supply is independent of season [11].

Numerous optimizations of lipase production have been studied over the past years since culture conditions have a huge impact on its production. In this situation, it may be beneficial to vary the components independently and investigate the effect of each component separately. However, such a process is inefficient both in terms of labor and accuracy [12]. Plackett-Burman design (PBD) is a statistical experimental design that is distinguished by its capacity to efficiently screen a large number of factors while minimizing the number of tests required [13]. Using the PBD, each factor was examined at two levels: -1 for low level and +1 for high level. The significance of this design is that it increases the efficiency of the experimental process by finding the most relevant components that affect activity, however, the outcome does not describe interactions among factors, and it is used to screen and evaluate factors for further optimization [12, 14].

Another approach, response surface methodology (RSM) which is a statistical tool for process optimization, is implemented to examine the interactions between factors. The most prevalent applications of RSM are in the industrial area, particularly when numerous input factors may influence the quality of a product or process [15]. RSM is an effective strategic technique that efficiently estimates the optimum conditions for a multivariable system by considering both the effects of the main components and their mutual interactions [13]. Furthermore, the RSM model can be validated through statistical tests to verify its reliability and accuracy, providing confidence in the optimal conditions.

Rajendran and Thangavelu studied the optimization of lipase production by *Candida rugosa* NCIM 3462 noting that the conventional approach, One-Factor-At-a-Time (OFAT), is time-consuming and its results may be misinterpreted. Therefore, a statistical approach, response surface methodology (RSM), was used to overcome this issue. The optimal medium obtained from this study was 19.604 g/l of glucose, 13.065 ml/l of groundnut oil, 7.473 g/l of peptone, 0.962 g/l of (NH_4_)_2_SO_4_ and 0.0019 g/l FeCl_3_·H_2_O yielding 5.95 U/ml of lipase activity [14]. Recently, optimization of lipase production by *Zygosaccharomyces mellis* SG1.2 was studied using the RSM method. The authors also suggested that statistical optimization is more advantageous than a conventional approach since it reduces the number of required experiments and evaluates the interaction effects between parameters. Additionally, an optimal medium contained 1.02% (v/v) of olive oil, 2.19% (w/v) of peptone, 0.05%(w/v) of MgSO_4_·7H_2_O, 0.05% (w/v) of KCl and 0.2% (w/v) of K_2_HPO_4_. It was found to increase lipase production by 1.8-fold compared to a basal medium [16].

Although lipase is in high demand and the conditions to improve its production have widely been investigated, the costly process of enzyme production has always been an obstacle to commercialization. The composition of the culture medium is very important for lipase production. Therefore, attempts to use inexpensive substrates have been continuously made to reduce production costs. The possibility of replacing expensive edible oils (olive oil) with non-edible oil waste (waste cooking oil, WCO) for lipase production was investigated using *Yarrowia lipolytica* and a doubled lipase activity (532 ± 21 U/l) was observed when olive oil was replaced by WCO. This raised interest from an economic standpoint. Additionally, 12,000 U/l lipase was produced in a stirred tank bioreactor using WCO as a low-cost substrate [17]. Cheese whey, the main by-product of cheese manufacturing, was also considered an alternative low-cost substrate for lipase production by *Meyerozyma guilliermondii* [18]. Cheese whey is the primary pollutant in dairy wastewater. An optimal result was found when cultivating yeast for 24 h in a medium containing 2.0% (w/v) cheese whey with pH adjusted to 4.0. A 6.7-fold increase in lipase production (285 U/ml) was achieved.

The search for alternative substrates that are low-cost but result in high yields is a clear requisite for all industrial enzyme and metabolite production. The present work, therefore, investigates an optimal medium for lipase production by *L. siamensis* DMKU-WBL1-3 using low-cost substrates to increase enzyme production. This is done by optimizing conditions for lipase production first at the flask level and later in a 2-L stirred-tank fermentor. Additionally, under optimal conditions for lipase production, this yeast was found to accumulate lipids inside the cells. The lipid content and its profile were also analyzed in the current study.

## Materials and Methods

### Yeast Strain Preservation

*Limtongozyma siamensis* DMKU-WBL1-3 was obtained from a grease sample and kept in the Department of Microbiology, Faculty of Science, Kasetsart University, Thailand. This yeast strain was cross-steaked on Yeast Extract Malt Extract (YM) agar (3 g/l of yeast extract, 3 of g/l malt extract, 5 of g/l peptone and 10 of g/l D-glucose) and incubated until colonies appeared, to assure purity. The purified yeast was preserved in a metabolically-inactive state (stored at −80°C in YM broth supplemented with 30% glycerol for long-term preservation).

### Optimization of Lipase Production

**Optimization of Medium Composition by a One-Factor-At-a-Time (OFAT) Approach**

The yeast strain was cultivated in YM medium for 18 h. Cell pellets were then collected, washed twice, resuspended in sterile distilled water, and used as an inoculum. The initial cell density was adjusted to 0.1 OD_600_. The experiment was conducted at 30°C for three days at 170 rpm on a rotary shaker. The supernatant was collected immediately after incubation to measure lipase activity using a titrimetric method [19].

The effect of different carbon sources on lipase production was investigated. This was done by replacing glucose with other carbon sources. Alternative carbon sources included lactose, starch, sucrose, and sweet whey. The carbon source with the greatest lipase production was chosen to assess the impact of different nitrogen sources in further experiments. Then, the nitrogen source was replaced by corn steep liquor, glutamate, peptone, skimmed milk, soy isolate, yeast extract (food grade) or yeast extract (technical grade).

Several types of commercially available oils were used in the medium to determine the effect of different oils on lipase production. These included palm, rice bran, soybean and sunflower oils. The base medium used in this experiment was YM medium supplemented with the most productive carbon and nitrogen sources. After sterilization of the base medium, 1% (v/v) of various oils was added using an aseptic technique and stored overnight at room temperature to assure sterility before use.

### Investigation of Significant Parameters Using Plackett-Burman Design (PBD) and Optimization of Lipase production Using Response Surface Methodology (RSM)

The Plackett-Burman design was used to screen the parameters affecting lipase production. Seven independent parameters, including inclusion in the media of sweet whey, yeast extract, malt extract, peptone, and oil, as well as adjustment of pH and inoculum size, were examined. Fifteen experimental runs were generated using Minitab Version 10.0.19044 to determine the responses. The effects of all factors on the responses were determined using analysis of variance (ANOVA). The parameters significantly impacting the responses were identified and optimized using response surface methodology (RSM) *i.e.*, Central Composite Design (CCD). Twenty-six CCD experiments were generated by the Design-Expert Version 13 Software Trial (Stat Ease Inc., USA). Similar to the PBD experiments, the effects of all factors on the responses were determined. Contour plots were drawn to depict optimal conditions for lipase production and to investigate the interactions between parameters. Validation experiments were conducted in accordance with the CCD.

To investigate the optimal time for lipase production, the incubation period was extended to nine days and samples were taken daily until the end of incubation. Lipase activity was immediately assayed after sampling. All experiments were performed in triplicate in 250 ml Erlenmeyer flasks containing 50 ml of medium and incubated at 30°C at 170 rpm on a rotary shaker.

### 2-L Stirred-Tank Fermentor Experiment

Several batch experiments were carried out in a 2-L stirred-tank fermentor (BIOSTAT, B. Braun Biotech International, Germany) with a 1.5 L working volume under optimal conditions. This was done to evaluate the effects of aeration and agitation on lipase production. A yeast inoculum was prepared in a 250 ml Erlenmeyer flask containing 30 ml of YM broth and incubated at 30°C for 18 h at 170 rpm on a rotary shaker. A 2% yeast inoculum (OD_600_ ≈ 5.0 – 5.2) was transferred into 1.47 L of the production medium. The fermenter was operated with varied aeration rates (1, 1.5, and 2 vvm) and agitation rates (170, 200, and 250 rpm). Samples were taken at the end of incubation followed by centrifugation at 4°C to collect the supernatant. Lipase activity was determined using a titrimetric method [16], whereas cell pellets were collected and washed twice with sterile distilled water before analysis of intracellular lipids.

### Determination of Lipase Activity

Lipase activity in the culture broth was determined using a titrimetric method based on olive oil hydrolysis [19]. A milliliter of supernatant was added to the assay substrate, which was composed of 10 ml of homogenized olive oil at 10% (v/v) in 10% (w/v) gum acacia, 2.0 ml of a 0.6% (v/v) CaCl_2_ solution, and 5 ml of phosphate buffer (pH 7.0). The enzyme-substrate mixture was incubated for one hour at 30°C and 150 rpm on a rotary shaker. After incubation, 20 ml of an alcohol and acetone mixture (1:1, freshly prepared) was added to the reaction solution. Liberated fatty acids were titrated with 0.1N NaOH using phenolphthalein as an indicator. The end-point color was light pink. Enzyme activity (U) was calculated as:

$$\text{Lipase activity=}\frac{\text{ΔV×N}}{\text{Vsample}}\text{×}\frac{\text{1000}}{\text{Incubation time }}$$

where ΔV = V_2_-V_1_, V_1_ is the volume of NaOH used for the control flask, V_2_ is the volume of NaOH used for the experimental flask, N is the normality of NaOH, and V_sample_ is the volume of supernatant. Incubation time was measured in minutes. Extracellular lipase activity was expressed as units per milliliter (U/ml). One unit of activity is defined as “the amount of enzyme which releases one micromole of fatty acid per minute under specified assay conditions”.

### Measurement of Yeast Growth

The growth parameter for lipase analysis was reported as the optical density (OD) of the culture. At the end of the incubation, one milliliter of culture was taken from the sample (the remaining culture was centrifuged at 4°C, and the supernatant was collected for lipase analysis). To avoid any possible inaccuracy in the optical density measurement caused by color intensity of the culture medium that contains different concentrations of sweet whey and palm oil in the CCD experiment. Hence, the sample was centrifuged, washed twice, and resuspended in distilled water. Cell suspension was then diluted until the optical density at 600 nm (OD_600_) was between 0.1 - 0.4 prior to multiplication by the dilution factor and the culture OD was obtained.

Instead of the OD_600_ measurement, yeast growth was determined as cell dry weight (CDW) in the experiment of lipid analysis. Cells were harvested by centrifugation at 8,200 ×*g* and 4°C after the completion of batch fermentation. The cell pellets were rinsed twice with distilled water before drying in the oven at 80°C until a constant weight was obtained.

### Measurement of Lipid Accumulation by Gas Chromatography (GC) Analysis

Lipid was extracted from dried cell pellets according to a modified method [20] followed by transmethylation of fatty acids to determine the fatty acid profile and lipid content of yeast cells [21]. Fatty acid methyl esters (FAMEs) were analyzed using gas chromatography (Shimazu, the Nexis GC-2030) with a capillary column (30 m × 0.32 mm × 0.25 μm, ZB-FFAP, Zebron). The column, injector and detector temperatures were 250, 210, and 250°C, respectively. Helium was used as a gas carrier. Fatty acids were identified and quantified by comparing their retention times and peak areas to those of standard fatty acids. Pentadecanoic acid (C_15:0_) was used as an internal standard. The sum of fatty acid concentrations per liter of culture broth and lipid content was reported as the total lipids per 100 grams of dry biomass (% of dry biomass).

## Results

The level of lipase production by *L. siamensis* DMKU-WBL1-3 depends on culture conditions and medium composition. Therefore, in this study, the effects of different carbon, nitrogen and oil sources were initially investigated using a One-Factor-At-a-Time (OFAT) approach. Once a suitable medium composition was obtained, the optimal level of each parameter was then evaluated using response surface methodology (RSM).

### Optimization of Lipase Production

**Optimization of Medium Composition Using a One-Factor-At-a-Time (OFAT) Approach**

**Fig. 1:**
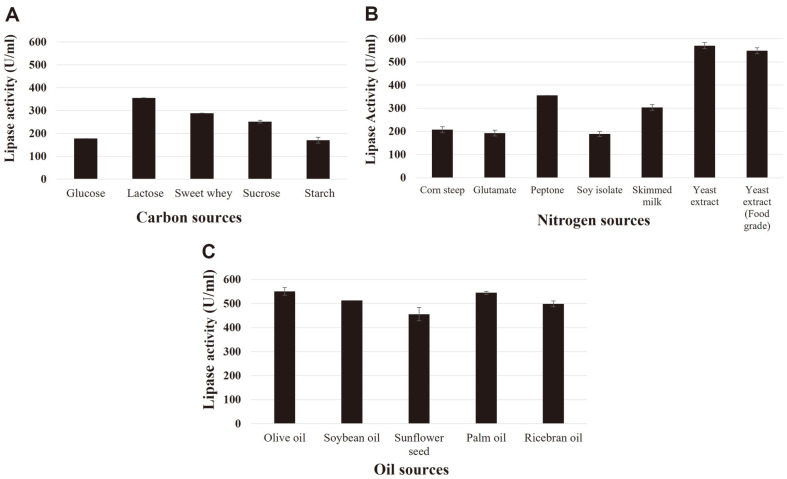
Effect of varying medium composition on lipase production determined by a One-Factor-At-a-Time (OFAT) approach. Different carbon sources (**A**), nitrogen sources (**B**), and alternative oil sources (**C**) were examined. The experiments were done in triplicate on rotary shaker at 30°C and 170 rpm for three days.

In the lipase production medium, glucose was replaced with other carbon sources to evaluate its impact on lipase production. The use of lactose as a carbon source resulted in the highest lipase production (355.6 ± 0.0 U/ml), followed by sucrose, glucose, and starch (251.8 ± 6.4, 177.8 ± 0.0, 170.4 ± 12.8 U/ml, respectively), as shown in Fig. 1A. However, in an effort to reduce production costs, sweet whey, which is high lactose (70%) by-product of the dairy industry [22], was used in place of glucose. The results of this experiment revealed a lipase production of 288.9 ± 0.0 U/ml. Even though lower lipase production was observed when sweet whey was used as a carbon source, its cost is lower than lactose making it a more economically valuable carbon source for lipase production (Table S1). However, there may be concerns about using sucrose which is cheaper than lactose, to reduce the cost of production. Table S1 indicates that, in comparison to sweet whey, sucrose shows a lower cost per gram but is more expensive in terms of cost per activity unit compared to lactose, sucrose, and sweet whey.

The effect of different nitrogen sources on lipase production was investigated. Yeast extract (technical grade) was found to be the best nitrogen source yielding 570.4 ± 12.8 U/ml lipase activity (Fig. 1B). Nevertheless, we also tried to further reduce production costs by replacing technical grade yeast extract with food grade yeast extract resulting in a lipase activity of 548.2 ± 12.8 U/ml, which is quite satisfactory. Hence, food-grade yeast extract was used in further experimentation.

Production of lipase by the yeast, *L. siamensis* DMKU-WBL1-3, requires oil as an inducer. Olive oil is commonly used for lipase production, but it is relatively expensive. Various types of commercial oils including palm, rice bran, soybean and sunflower oils were investigated as low-cost inducers. The results showed lipase production of 545.1 ± 16.4 U/ml and 550.8 ± 16.0 U/ml were obtained when palm oil and olive oil were used, respectively (Fig. 1C). Palm oil was therefore selected for further experimentation. Using an OFAT approach, it was concluded that sweet whey, yeast extract (food grade), and palm oil can serve as carbon, nitrogen, and oil sources, respectively, for lipase production by *L. siamensis* DMKU-WBL1-3.

### Investigation of Significant Parameters Using Plackett-Burman Design (PBD) and Optimization of Lipase Production Using Response Surface Methodology (RSM)

To study parameters affecting lipase production by *L. siamensis* DMKU-WBL1-3, media components as well as pH and inoculum size were examined using Plackett-Burman design (PBD). Based on the ANOVA results, a model obtained from the PBD experiment was significant (*p*-value < 0.05) with an R^2^ value of 0.9280. This indicates that the model can be used to explain 92.8% of the variability in the responses. The significant factors for lipase production were sweet whey, yeast extract, malt extract, palm oil levels and pH whereas peptone level and inoculum size had no effect (Table 1). Thus, the five significant factors (sweet whey, yeast extract, malt extract, palm oil and pH) were further optimized using central composite design (CCD).

**Fig. 2:**
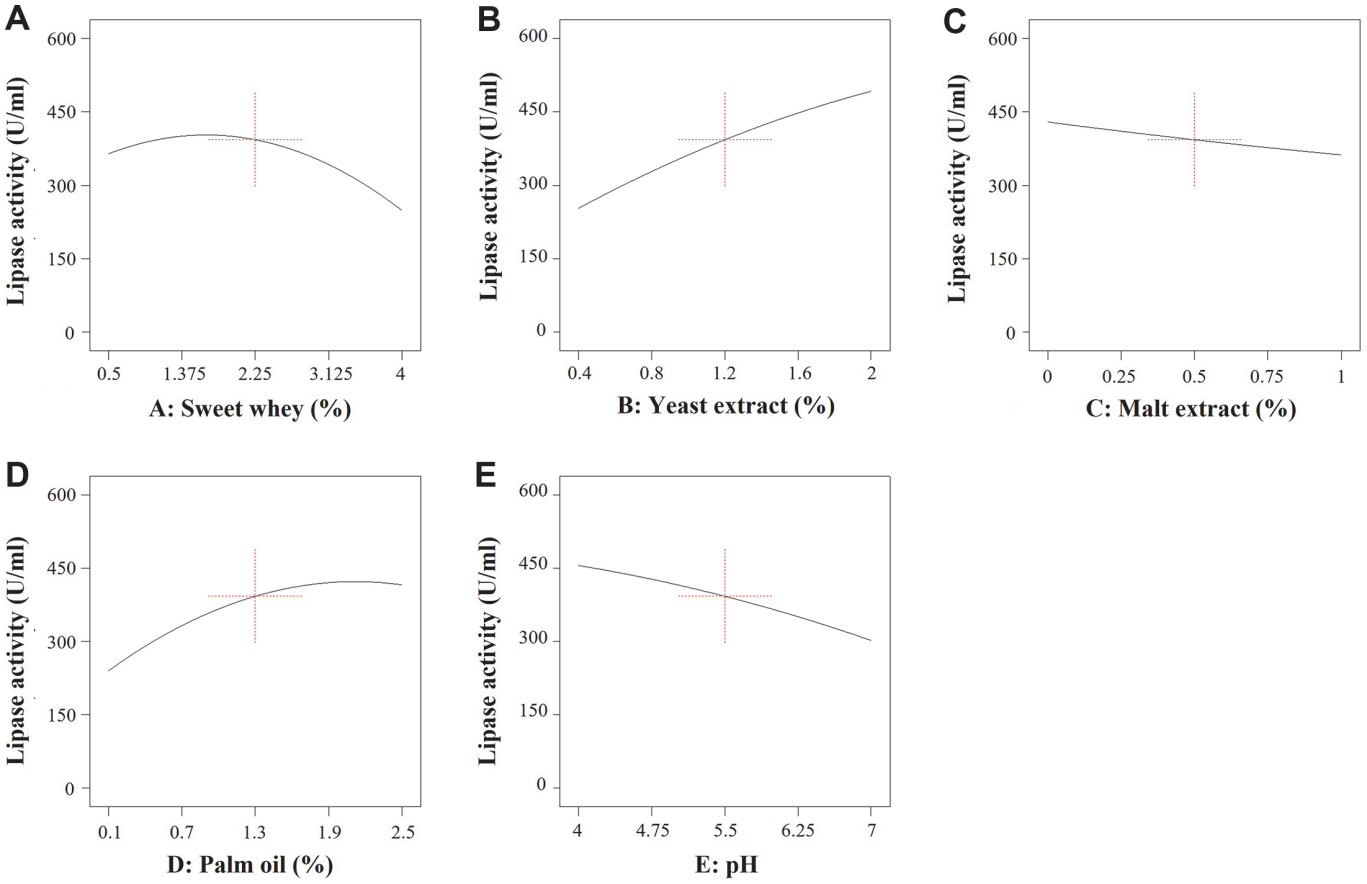
The individual effects of sweet whey (A), yeast extract (B), malt extract (C), palm oil (D), and pH (E) on lipase production by *L. siamensis* DMKU-WBL1-3. The Y-axis represents the average lipase production on each level and the X-axis indicates the concentration of each parameter studied (percent units for parameters A-D and level units for parameter E).

Twenty-six experiments were designed using the Design-Expert program. Lipase production ranged from 3.7 ± 0.0 to 546.3 ± 16.0 U/ml, as shown in Table 2. The individual effects of each parameter are revealed in Fig. 2. The results are shown in Fig. 2A. indicated that the highest region represented the optimal concentration of sweet whey (approximately 0.5–1.375%). Greater sweet whey concentrations showed a negative effect on lipase production. Increased yeast extract concentrations elevated lipase production (Fig. 2B). However, the opposite result was found for malt extract. Lipase production slightly decreased with increased malt extract concentration (Fig. 2C). Furthermore, increased palm oil concentration had a positive effect up to a 2.5% level. Thereafter, lipase production remained stable (Fig. 2D) while increasing pH showed a negative result (Fig. 2E).

**Fig. 3:**
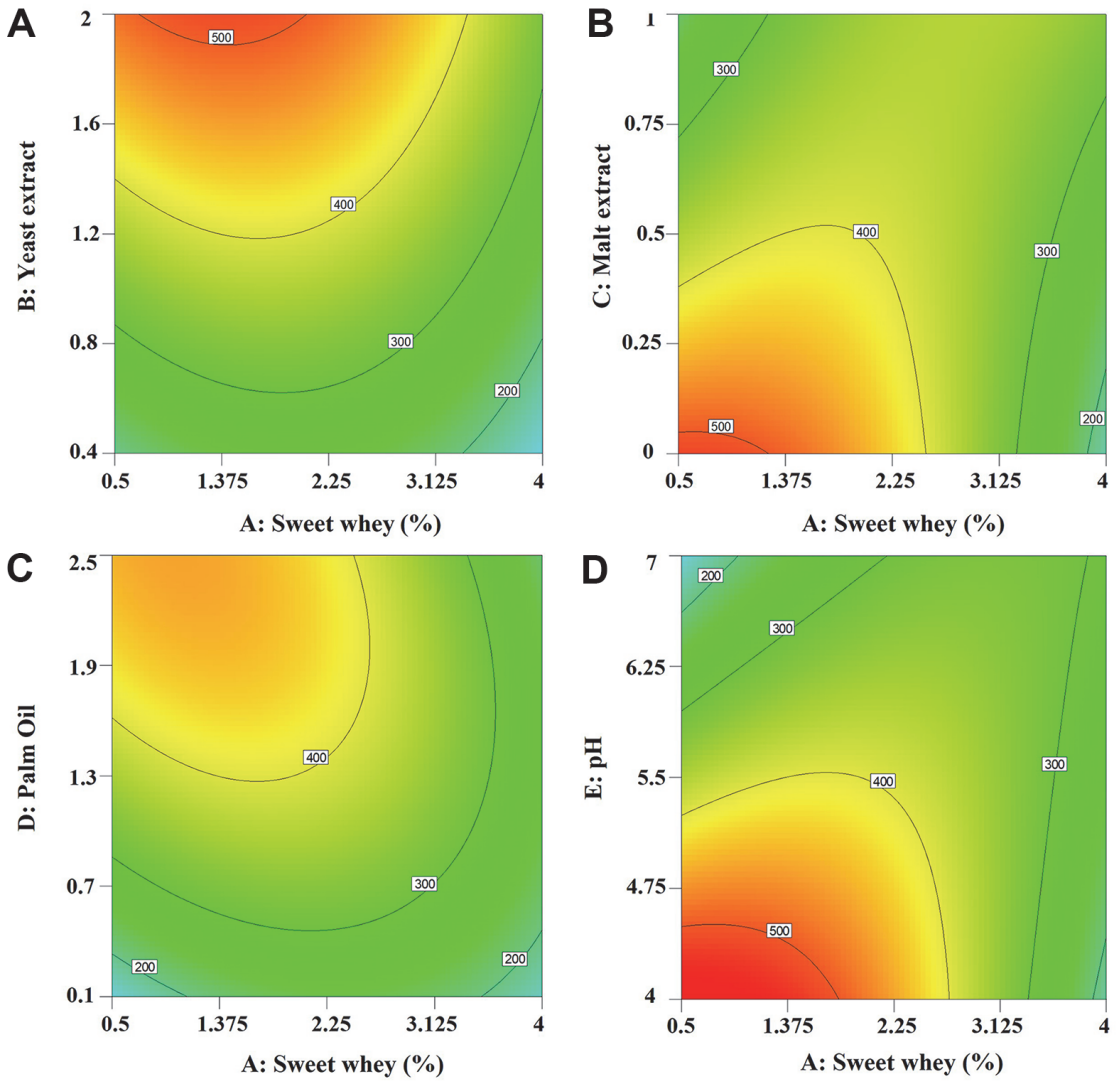
Response surface plots described by the model for lipase production by *L. siamensis* DMKU-WBL1-3, representing the interactive effects between parameters, sweet whey with yeast extract (A), sweet whey with malt extract (B), sweet whey with palm oil (C), and sweet whey with pH (D). The Y-axis represents the average of lipase production on each level while the X-axis indicates the concentration of each parameter studied (percent units for parameters A-D and level units for parameter E).

Fig. 3. shows contour plots depicting the interactions between sweet whey and other parameters when the concentration of sweet whey was kept between set values. The contour plots in Fig. 3A. indicate that lipase production approached its peak when the sweet whey concentration remained in the range of 0.5–2.25%. Additionally, yeast extract concentration was directly proportional to lipase production (Fig. 3A). A similar pattern of response was shown between the interaction of palm oil and sweet whey for lipase production (Fig. 3C). However, the oil concentration needs to be controlled as oils have been reported to increase the medium viscosity which impacts oxygen transmission [23]. Enzyme production increased with decreased malt extract and pH levels (Figs. 3B and 3D). However, it is notable that an excessively low pH level may result in an acidic medium in which yeast cannot grow.

The ANOVA results show that the model for lipase production had a *p* > *F*-value of 0.0001, indicating that the model is significant for all responses and an R^2^ value of 0.9991 indicating that the model can explain 99.91% of the variation in the responses. Validation experiments were conducted, and the results are shown in Table 3. The error between the predicted and actual values from the validation experiment was less than 5%. The final response equation for lipase production is:

Lipase activity = +393.07171 - 57.96061*A + 119.48019*B - 33.55614*C + 87.95776*D - 76.77238*E - 27.74250*AB + 114.18272*AC - 63.81266*AD + 134.25082*AE + 27.94562*BC - 200.04976*BD - 8.00480*BE - 47.93935*CD + 144.10561*CE - 107.03792*DE - 86.61643* A2 - 20.45144*B2 + 2.72027*C2 - 64.56143*D2 - 13.75118*E2

where A is sweet whey concentration, B is yeast extract (food grade) concentration, C is malt extract concentration, D is palm oil concentration and E is pH level.

**Fig. 4:**
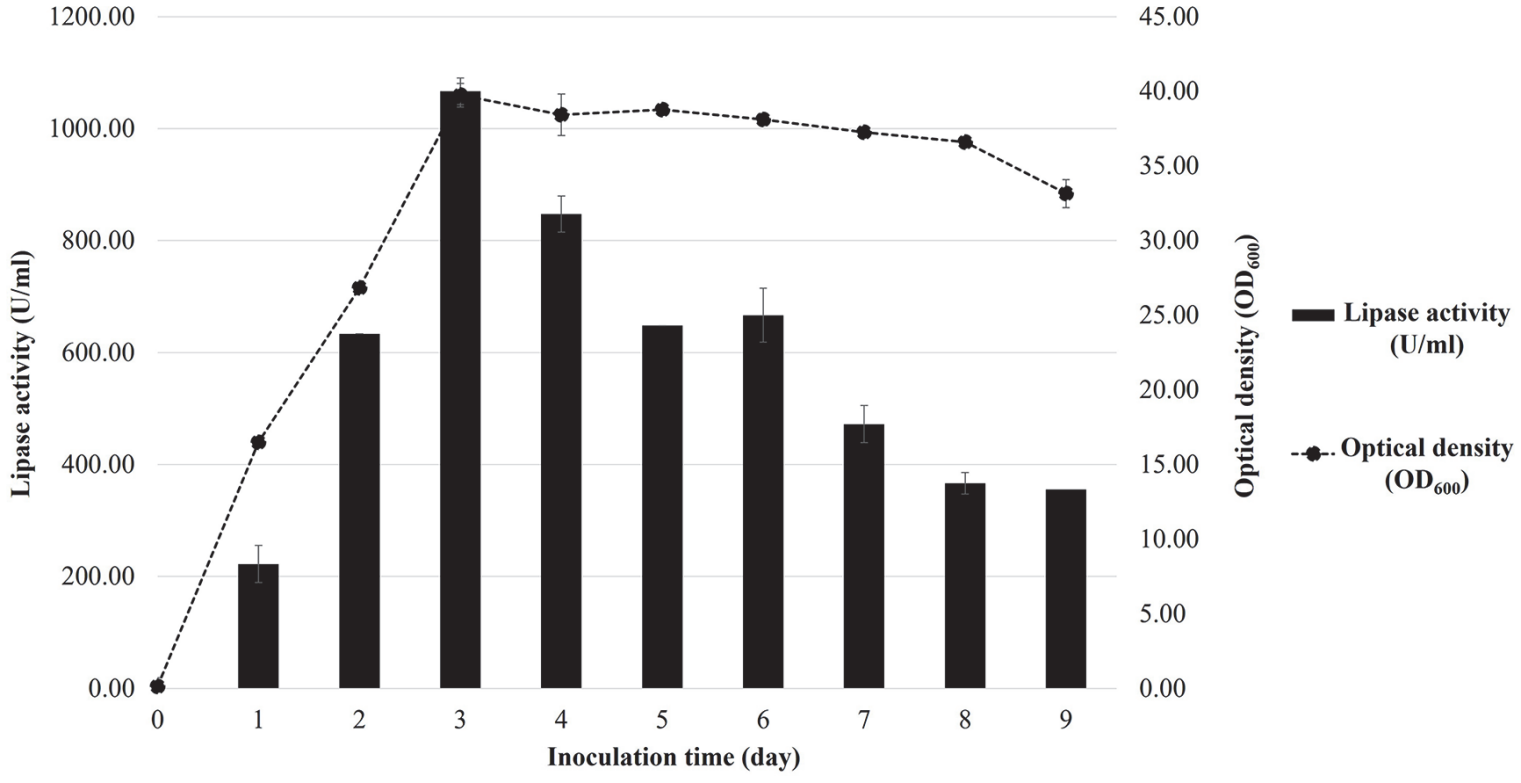
Time-course experiment of lipase production by *L. siamensis* DMKU-WBL1-3.

This model is suitable to predict the optimal medium for lipase production by *L. siamensis* DMKU-WBL1-3. The results of the validation experiments are shown in Table 3. The greatest lipase production was found in Run No. 1 (1,327.8 ± 23.6 U/ml). However, the medium costs of Run No. 1, 2, 3 and 4 are 0.64, 0.64, 0.11 and 0.37 US dollars (USD) per liter, respectively (Table 4). Therefore, when considering cost reduction, Run No. 3 showed the best potential as a suitable low-cost medium for lipase production since it is 87.5% less expensive than the original medium (Table S2). Hence, a suitable low-cost medium for *L. siamensis* DMKU-WBL1-3 lipase production was that obtained in Run No. 3 which comprised 0.50% (w/v) sweet whey, 0.40% (w/v) yeast extract (food grade), and 2.50% (v/v) palm oil with pH adjusted to 4.0. After obtaining the optimal medium, a time-course experiment was carried out. Its results revealed that yeast grew rapidly (entered an exponential phase) from Day 0 to Day 3 without a lag phase of growth. Lipase production was also observed (Fig. 4). The optimal incubation time for lipase production is three days, as indicated by the highest biomass and lipase production.

### 2-L Stirred-Tank Fermentor Experiment

A batch cultivation process was conducted in a 2-L stirred-tank fermentor to evaluate the influence of aeration rate and agitation rate on lipase production. The experiments were performed at three different aeration rates, 1, 1.5 and 2 vvm, while the agitation rate and incubation temperature were maintained constant at 170 rpm and 30 ± 2°C, respectively. Lipase activities were found to be 1,055.6 ± 0.0, 965.3 ± 68.8, and 847.2 ± 58.9 U/ml when the aeration rate was adjusted to 1, 1.5, and 2 vvm, respectively. It was found that increasing the aeration rate leads to the formation of cell clumps and decreased lipase production. This is consistent with previous reports on low lipase production at high dissolved oxygen contents [24] and cell clumping [11]. In the present study, we also found that an increase in agitation speed decreased the occurrence of cell clumps due to centrifugal force from the impeller. We therefore postulated that not only was agitation responsible for the formation of clumps, but also a high aeration rate (2 vvm). Nevertheless, aeration rates have different effects on various organisms [25]. So, it is notable that an increased aeration rate may result in greater lipase production in other yeast species. However, in the present research, the production of lipase decreased with increased aeration and the optimal aeration rate in this study was 1 vvm.

At the constant aeration rate of 1 vvm, the effect of the agitation rate was investigated at 170, 200, and 250 rpm. Increasing the agitation rate to 200 and 250 rpm decreased lipase production and cell dry weight (Table 5). This may have been due to increased oxidative or shear stresses, which led to decreased yeast growth and lipase production [26].

Mechanical shear stress influences the morphology, metabolism, and viability of yeast cells. The effect of shear stress on lipid production by *Rhodotorula mucilagenosa* has been reported. The author proposed that shear stress thresholds of 0.22-68 Pa had no hazardous effect on the yeast cells [27]. Interestingly, *S. cerevisiae* has been shown to be extremely resistant to shear stress, with thresholds of more than 1,292 Pa resulting in non-significant viability loss [28]. It should be emphasized, however, that Basidiomycetes are more sensitive to shear stress than Ascomycetes [29]. Aside from that, there was a report on inulinase production by *Kluyveromyces marxianus* at which shear stress was determined to be 0.12, 0.219, and 0.333 Pa at agitation rates of 100, 150, and 200 rpm, respectively. Shear stress had less effect on inulinase production at 150 rpm, but at 200 rpm, it caused a decrease in biomass and product concentration [30]. This study showed that *K. marxianus* was more sensitive to shear stress than *S. cerevisiae*. In our study, *L. siamensis* DMKU-WBL1-3 exhibits similar shear stress sensitivity to that of the previously mentioned *K. marxianus*. Increasing the agitation rate resulted in a drop in cell dry weight. We hypothesize that increasing the agitation rate has both positive and negative effects on the cells. The positive effect is that the cells could effectively contact the medium, helping them to better produce enzymes and adsorb fatty acids from the environment. Meanwhile, an increase in shear stress also emerged when the agitation rate increased. Aside from causing physical harm to the cells, raising the agitation rate increases the dissolved oxygen in the system, which inhibits lipase production and lipid accumulation as well.

Based on our findings, the suitable aeration and agitation rates for *L. siamensis* DMKU-WBL1-3 lipase production in a 2-L stirred-tank fermentor are 1 vvm and 170 rpm, respectively. The minimum aeration rate was set to 1 vvm due to the limitations of the fermentor controller. The minimum agitation rate was set at 170 rpm because preliminary experiments performed in flasks revealed that reduced agitation rates resulted in lower yeast growth, which in turn resulted in decreased enzyme production (Fig. S1).

### Lipid Accumulation and Lipid Profile

**Fig. 5:**
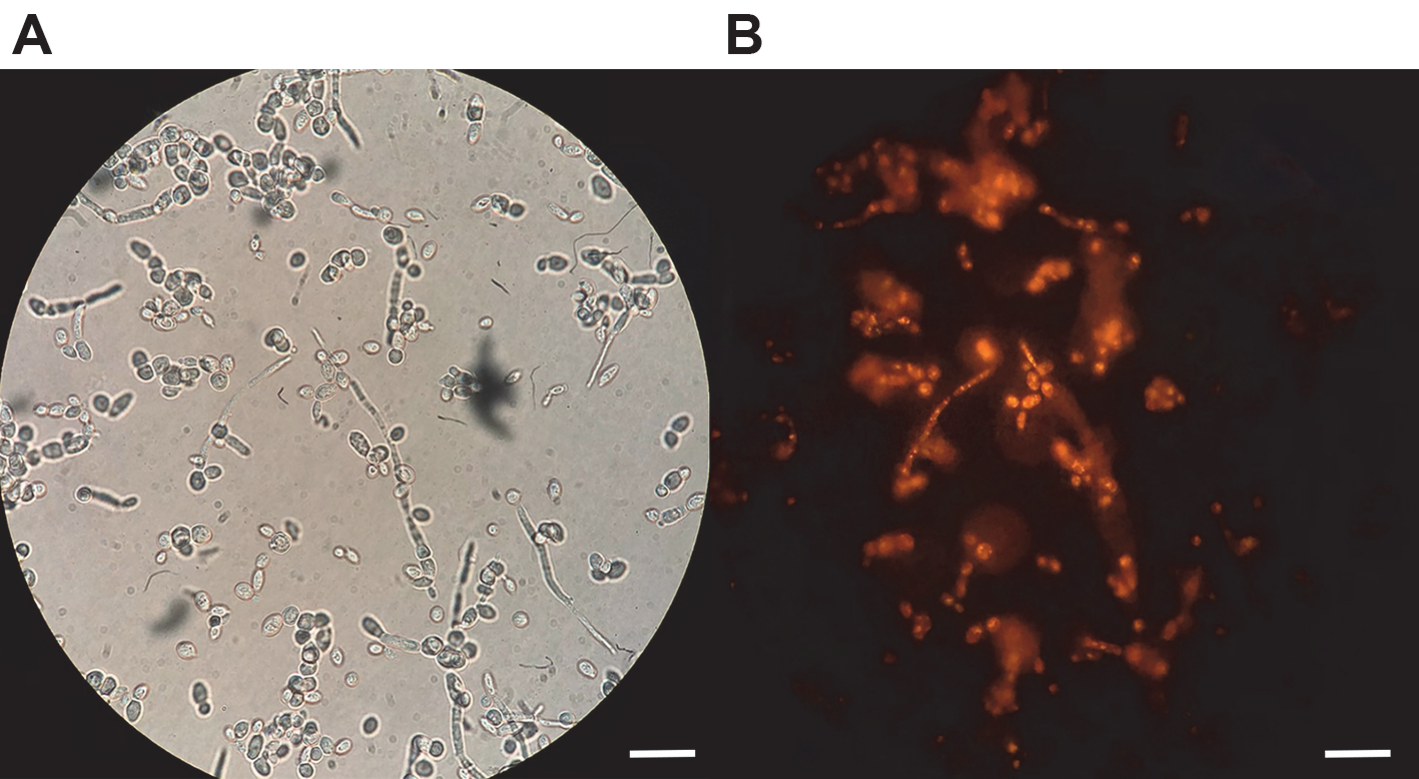
Imagery of Nile red-stained cells of *L. siamensis* DMKU-WBL1-3 cultivated in a 2-L fermenter under optimal conditions for three days. The bar is 10 μm long, (**A**) bright-field image, (**B**) fluorescence microscopy image.

Aside from lipase-producing ability, previous studies revealed that lipid accumulation is a parallel trait present in lipase-producing yeasts [17, 31, 32]. The presence of lipases in the environment enables many yeasts to consume lipids or other substances with ester linkages as carbon sources and absorb them back into the cells for storage as reserve energy. In this study, *L. siamensis* DMKU-WBL1-3 also showed the capability to accumulate intracellular lipids (Table 6, Fig. 5). The results obtained in this study were consistent with a previous study reporting that high aeration and agitation rates decreased lipid accumulation [33] and possibly hastened its decomposition by increasing acyl CoA oxidase activity [34]. Additionally, there was a report that the highest microbial oil accumulation was obtained when the medium dissolved oxygen concentration remained nearly zero [17].

The main fatty acids accumulated by *L. siamensis* DMKU-WBL1-3 were palmitoleic (C_16:1_) and oleic (C_18:1_) acids, which are monounsaturated fatty acids (MUFAs), as shown in Table 7. Since unsaturated fatty acids are typically linked to health benefits [17], the fatty acids accumulated in this yeast are therefore of interest for further study. However, the fatty acid content of a substrate greatly influences its microbial uptake and oil composition. Cultivating this yeast on other oil sources or in other media may reveal different intracellular fatty acid compositions.

## Discussion

Lipase is an enzyme that is used in many industries due to its wide range of applications. It can be produced using several processes including solid state fermentation by filamentous fungi and submerged fermentation by yeast and bacteria. However, submerged cultivation of yeast has been found to be the most suitable process for the production of lipase [11]. As previously stated, process costs remain one of the most significant issues in industrial lipase production. Therefore, the present work was carried out to demonstrate lipase production by *L. siamensis* DMKU-WBL1-3, a yeast strain isolated from grease waste, using low-cost substrates to mitigate lipase production costs. It was found that this yeast has the potential to produce 1055.6 ± 0.0 U/ml of lipase in a fermentor when cultivated for three days in a medium employing low-cost substrates making it 87.5% less expensive than the original medium (Table S2). However, other investigations using the same titrimetric method showed higher lipase production levels [35, 36], but the results are not comparable because different culture conditions and yeasts were studied.

Our findings highlight the potential of *L. siamensis* DMKU-WBL1-3. Under optimal lipase production by this yeast, lipid accumulation was also observed, although no large amount of intracellular lipid was found. The primary fatty acids accumulated by *L. siamensis* DMKU-WBL1-3 were monounsaturated fatty acids (MUFAs) typically linked to health benefits. Further optimization may be conducted to promote lipid accumulation in this yeast both in terms of amount and type of lipids for wider applications such as in the food industry or for biofuels. Therefore, it is important to continue research to quantify the relationship between lipase production and intracellular lipid accumulation by this yeast.

## Supplemental Materials

Supplementary data for this paper are available on-line only at http://jmb.or.kr.



## Figures and Tables

**Table 1:** Analysis of variance (ANOVA) results of Plackett-Burman design (PBD) for parameters affecting lipase production by *Limtongozyma siamensis* DMKU-WBL1-3 incubated at 30°C and 170 rpm for three days.

Source	*F*-Value	*p*-Value
Model	9.67	0.006
Sweet whey	17.54	0.006
Yeast extract	8.01	0.030
Malt extract	6.70	0.041
Peptone	1.95	0.212
Oil	9.84	0.020
pH	13.01	0.011
Inoculum size	5.63	0.055
Lack-of-Fit	Not sig	Not sig

**Table 2:** RSM-CCD experiments designed by Design-Expert program with actual and predicted values of lipase production and optical density (OD_600_) of *Limtongozyma siamensis* DMKU-WBL1-3 incubated at 30°C and 170 rpm for three days.

Run	Sweet whey (% w/v)	Yeast extract (% w/v)	Malt extract (% w/v)	Palm oil (% v/v)	pH	Lipase activity (U/ml)	Optical density (OD_600_)
1	4.00	2.00	0.00	2.50	4.0	207.4	36.27
2	4.00	0.40	1.00	2.50	4.0	185.2	35.64
3	0.50	2.00	1.00	0.10	7.0	422.2	7.26
4	4.00	2.00	1.00	0.10	4.0	274.1	33.91
5	4.00	2.00	0.00	0.10	7.0	274.1	33.49
6	4.00	0.40	0.00	2.50	7.0	92.6	31.78
7	0.50	0.40	1.00	2.50	7.0	81.5	41.36
8	0.50	2.00	0.00	2.50	7.0	63.0	51.21
9	4.00	0.40	1.00	0.10	7.0	251.9	33.28
10	0.50	2.00	1.00	2.50	4.0	379.6	40.26
11	0.50	0.40	0.00	0.10	4.0	133.3	4.94
12	-0.94	1.20	0.50	1.30	5.5	214.8	28.20
13	5.44	1.20	0.50	1.30	5.5	3.7	35.29
14	2.25	-0.26	0.50	1.30	5.5	111.1	17.94
15	2.25	2.66	0.50	1.30	5.5	546.3	34.03
16	2.25	1.20	-0.410	1.30	5.5	466.7	32.61
17	2.25	1.20	1.41	1.30	5.5	344.4	32.38
18	2.25	1.20	0.50	-0.890	5.5	22.2	6.42
19	2.25	1.20	0.50	3.49	5.5	342.6	60.32
20	2.25	1.20	0.50	1.30	2.8	490.7	22.52
21	2.25	1.20	0.50	1.30	8.2	211.1	34.58
22	2.25	1.20	0.50	1.30	5.5	398.2	33.23
23	2.25	1.20	0.50	1.30	5.5	385.2	31.24
24	2.25	1.20	0.50	1.30	5.5	377.8	32.91
25	2.25	1.20	0.50	1.30	5.5	400.0	31.36
26	2.25	1.20	0.50	1.30	5.5	392.6	30.44

**Table 3:** Validation experiments with actual and predicted values of lipase production.

Run	Sweet whey (% w/v)	Yeast extract (% w/v)	Malt extract (% w/v)	Palm oil (% v/v)	pH	Lipase activity (U/ml)		
						Pred. ^a^	Act. ^b^	Error (%) ^c^
1	0.90	4.00	0.00	0.10	4.0	1337.5	1327.8	0.73
2	0.50	4.00	0.00	0.10	4.0	1380.1	1314.8	4.73
3	0.50	0.40	0.00	2.50	4.0	1150.8	1105.6	3.93
4	0.50	2.00	0.00	2.50	4.0	1005.2	1041.7	3.63

^a^Predicted value from the software output.

^b^Actual value from the experiment

^c^(Difference between the predicted and actual value / predicted value) × 100

**Table 4:** Comparison of medium cost in validation experiments and original medium with a total cost of 0.85 USD/l (the original medium consisted of lactose, yeast extract (technical grade), malt extract, and olive oil).

Run	Cost of medium components* (USD/l)			Total cost of medium** (USD/l)
	Sweet whey	Yeast extract (Food grade)	Palm oil	
1	0.01	0.63	0.001	0.64
2	0.01	0.63	0.001	0.64
3	0.01	0.06	0.04	0.11
4	0.01	0.32	0.04	0.37

*Cost of medium components are as follows: Sweet whey 0.001 USD/g ([22]), Yeast extract (food grade) 0.016 USD/g (Thai food international Co., Ltd., Thailand), Palm oil 0.001 USD/g (Commercial oil from a supermarket.)

**The cost calculation of the original medium is a cost comparison between the original substrates and alternative substrates using the optimal medium concentration (Run 3). USD = 33.714 THB (Exchange rate on May 9, 2023)

**Table 5:** Lipase activity, dry biomass, and lipid accumulation by *Limtongozyma siamensis* DMKU-WBL1-3 in a 2-L stirred-tank fermentor.

Condition	Lipase activity (U/ml)	Dry biomass (g/l)	Total lipid (g/l)	Lipid content (%)
170 rpm, 30°C, 1 vvm	1055.6 ± 0.0	12.32 ± 0.03	3.79 ± 0.03	30.8
170 rpm, 30°C, 1.5 vvm	965.3 ± 68.8	17.40 ± 0.20	2.24 ± 0.03	12.9
170 rpm, 30°C, 2 vvm	847.2 ± 58.9	16.06 ± 0.73	1.49 ± 0.07	9.28
200 rpm, 30°C, 1 vvm	777.1 ± 40.3	10.23 ± 0.30	0.93 ± 0.01	9.1
250 rpm, 30°C, 1 vvm	781.2 ± 24.6	8.42 ± 0.78	1.04 ± 0.00	12.4

**Table 6:** The retention time between standard fatty acids and samples.

Batch 1						
Standard fatty acids	Time (Min.)			Peak area		
	Standard	Replicate 1	Replicate 2	Standard	Replicate 1	Replicate 2
14:0	3.64	3.64	3.64	90266	1011	1001
15:0	4.48	4.48	4.48	27498	27534	27072
16:0	5.47	5.44	5.44	38469	5949	4940
16:1	5.68	5.67	5.68	69549	79718	78923
18:0	9.00	8.98	9.00	96368	43973	42706
18:1	9.13	9.12	9.17	307615	435583	427902
18:2	9.28	9.25	9.27	320403	23095	20825
18:3G	9.75	9.73	9.74	48292	20901	20055
18:3A	14.46	14.46	14.46	40794	1041	4301
Batch 2						
Standard fatty acids	Time (Min.)			Peak area		
	Standard	Replicate 1	Replicate 2	Standard	Replicate 1	Replicate 2
14:0	3.64	3.64	3.64	99322	1446	1305
15:0	4.48	4.48	4.48	29293	29214	28220
16:0	5.46	5.45	5.44	41668	4858	4602
16:1	5.67	5.68	5.68	76034	88952	83257
18:0	8.99	9.00	9.00	108023	49837	45906
18:1	9.13	9.18	9.17	341155	510121	470171
18:2	9.28	9.27	9.27	387669	26018	22087
18:3G	9.74	9.75	9.74	54362	23102	20978
18:3A	14.45	14.47	14.46	44578	19504	25986

**Table 7:** Profile of fatty acids accumulated by *Limtongozyma siamensis* DMKU-WBL1-3 after three days incubation in a 2-L stirred-tank fermentor under various conditions.

Condition	Total lipid (g/l)	Percent of fatty acids composition							
		14:0	16:0	16:1	18:0	18:1	18:2	18:3G	18:3A
170 rpm, 30°C, 1 vvm	3.75	0.32	3.44	30.72	12.06	38.38	1.76	11.20	2.12
170 rpm, 30°C, 1.5 vvm	2.24	0.00	3.20	30.54	11.99	38.42	1.61	11.87	2.38
170 rpm, 30°C, 2 vvm	1.49	0.00	2.99	31.82	11.07	33.82	2.73	12.09	5.48
200 rpm, 30°C, 1 vvm	0.93	0.00	6.54	38.74	8.19	19.00	7.60	8.55	11.40
250 rpm, 30°C, 1 vvm	1.04	0.00	3.08	30.29	9.21	30.34	3.92	11.48	11.68

## References

[1] Boontham W, Angchuan J, Boonmak C, Srisuk N (2020). *Limtongozyma siamensis* gen. nov., sp. nov., a yeast species in the Saccharomycetales and reassignment of *Candida cylindracea* to the genus *Limtongozyma*. Int. J. Syst. Evol. Microbiol..

[2] Zalacain I, Zapelena MJ, Astiasarán I, Bello J (1995). Dry fermented sausages elaborated with lipase from *Candida cylindracea* comparison with traditional formulations. Meat Sci..

[3] Kim BS, Hou CT (2006). Production of lipase by high cell density fed-batch culture of *Candida cylindracea*. Bioprocess Biosyst. Eng..

[4] Noor I, Hasan M, Ramachandran K (2006). Effect of carbon and nitrogen sources on the production of lipase by *Candida cylindracea* 2031 in Batch Fermentation. J. Environ. Chem. Eng..

[5] Belkacemi FZ, Merabet-Khelassi M, Aribi-Zouioueche L, Riant O (2018). Production of l-menthyl acetate through kinetic resolution by *Candida cylindracea* lipase: effects of alkaloids as additives. Res. Chem. Intermed..

[6] Tang A, Zhang Y, Wei T, Wu J, Li Q, Liu Y (2019). Immobilization of *Candida cylindracea* lipase by covalent attachment on glumodified bentonite. Appl. Biochem. Biotechol..

[7] Zieniuk B, Mazurczak-Zieniuk P, Fabiszewska A (2020). Exploring the impact of lipid-rich food industry waste carbon sources on the growth of *Candida cylindracea* DSM 2031. Fermentation.

[8] Yan J, Yan Y, Madzak C, Han B (2017). Harnessing biodiesel-producing microbes: from genetic engineering of lipase to metabolic engineering of fatty acid biosynthetic pathway. Crit. Rev. Biotechnol..

[9] Sarmah N, Revathi D, Sheelu G, Yamuna Rani K, Sridhar S, Mehtab V (2018). Recent advances on sources and industrial applications of lipases. Biotechnol. Progr..

[10] Huang AHC, Brockman BHL (1984). Plant lipases. Lipases.

[11] Krastanov A, Govindarajan A, Daniel D (2008). Studies on lipase fermentation using *Candida cylindracea* NRRL Y-17506 in a stired tank bioreactor. Bulg. J. Agric. Sci..

[12] Plackett RL, Burman JP (1946). The design of optimum multifactorial experiments. Biometrika.

[13] Mandal S, Bhunia B, Kumar A, Dasgupta D, Mandal T, Datta S (2013). A statistical approach for optimization of media components for phenol degradation by *Alcaligenes faecalis* using Plackett-Burman and response surface methodology. Desilin. Water Treat..

[14] Rajendran A, Thangavelu V (2007). Optimization of medium composition for lipase production by *Candida rugosa* NCIM 3462 using response surface methodology. Can. J. Microbiol..

[15] Myers RH, Montgomery DC, Anderson-Cook CM (2009). Response Surface Methodology : Process and Product Optimization Using Designed Experiments..

[16] Pramitasari MD, Ilmi M (2021). Optimization of medium for lipase production from *Zygosaccharomyces mellis* SG1.2 using statistical experiment design. Microbiol. Biotechnol. Lett..

[17] Lopes M, Miranda SM, Alves JM, Pereira AS, Belo I (2018). Waste cooking oils as feedstock for lipase and lipid‐rich biomass production. Eur. J. Lipid Sci. Technol..

[18] Knob A, Izidoro SC, Lacerda LT, Rodrigues A, de Lima VA (2020). A novel lipolytic yeast *Meyerozyma guilliermondii*: efficient and low-cost production of acid and promising feed lipase using cheese whey. Biocatal. Agric. Biotechnol..

[19] Pandey S, Srivastava M, Shahid M, Kumar V, Singh A, Trivedi S (2016). Isolation, production and partial characterization of extracellular lipase from *Trichoderma* species. J. Pure. Appl. Microbiol..

[20] Blight EG, Dyer WJ (1959). A rapid method for total lipid extraction and purification. Can. J. Biochem. Physiol..

[21] Holub BJ, Skeaff CM (1987). Nutritional regulation of cellular phosphatidylinositol. Methods Enzymol..

[22] Srisuk N, Sakpuntoon V, Nutaratat P (2018). Production of indole-3-acetic acid by *Enterobacter* sp. DMKU-RP206 using sweet whey as a low-cost feed stock. J. Microbiol. Biotechnol..

[23] Tamilarasan (2011). Optimization of medium components and operating conditions for the production of solvent-tolerant lipase by *Bacillus sphaericus* MTCC 7542. Afr. J. Biotechnol..

[24] Puthli MS, Rathod VK, Pandit AB (2006). Optimization of lipase production in a triple impeller bioreactor. Biochem. Eng. J..

[25] Ghosh PK, Saxena RK, Gupta R, Yadav RP, Davidson S (2016). Microbial lipases: production and applications. Sci. Prog..

[26] Snopek P, Nowak D, Zieniuk B, Fabiszewska A (2021). Aeration and stirring in *Yarrowia lipolytica* lipase biosynthesis during batch cultures with waste fish oil as a carbon source. Fermentation.

[27] Banerjee A, Sharma T, Nautiyal AK, Dasgupta D, Hazra S, Bhaskar T (2020). Scale-up strategy for yeast single cell oil production for *Rhodotorula mucilagenosa* IIPL32 from corn cob derived pentosan. Bioresour. Technol..

[28] Lange Hln, Taillandier P, Riba J-P (2001). Effect of high shear stress on microbial viability. J. Chem. Technol. Biotechnol..

[29] Jonczyka P, Takenbergb M, Hartwiga S, Beutel S, Berger RG, Thomas S (2013). Cultivation of shear stress sensitive microorganisms in disposable bag reactor systems. J. Biotechnol..

[30] Santharam L, Easwaran SN, Subramanian Mohanakrishnan A, Mahadevan S (2019). Effect of aeration and agitation on yeast inulinase production: a biocalorimetric investigation. Bioprocess. Biosyst. Eng..

[31] Sakpuntoon V, Angchuan J, Boontham W, Khunnamwong P, Boonmak C, Srisuk N (2019). Grease waste as a reservoir of lipaseproducing yeast and description of *Limtongella siamensis* gen. nov., sp. nov. Microorganisms.

[32] Ayadi I, Belghith H, Gargouri A, Guerfali M (2018). Screening of new oleaginous yeasts for single cell oil production, hydrolytic potential exploitation and agro-industrial by-products valorization. Process Saf. Environ. Prot..

[33] Rakicka M, Lazar Z, Dulermo T, Fickers P, Nicaud JM (2015). Lipid production by the oleaginous yeast *Yarrowia lipolytica* using industrial by-products under different culture conditions. Biotechnol. Biofuels..

[34] Papanikolaou S, Aggelis G (2011). Lipids of oleaginous yeasts. Part I: Biochemistry of single cell oil production. Eur. J. Lipid Sci. Technol..

[35] Kamzolova SV, Morgunov IG, Aurich A, Perevoznikova OA, Shishkanova NV, Stottmeister U (2005). Lipase secretion and citric acid production in *Yarrowia lipolytica* yeast grown on animal and vegetable fat. Food Technol. Biotechnol..

[36] Turki S, Ayed A, Chalghoumi N, Weekers F, Thonart P, Kallel H (2010). An enhanced process for the production of a highly purified extracellular lipase in the non-conventional yeast *Yarrowia lipolytica*. Appl. Biochem. Biotechnol..

