# Supplementary material for: Lipase Production by Limtongozyma siamensis, a Novel Lipase Producer and Lipid Accumulating Yeast
Source: J Microbiol Biotechnol. 2023 Jul 10;33(11):1531–41. doi: 10.4014/jmb.2304.04006 (PMC10699263; doi:10.4014/jmb.2304.04006)
Supplement: Supplementary file 1 [file jmb-33-11-1531-supple.pdf]

J. Microbiol. Biotechnol.
https://doi.org/10.4014/jmb.2304.04006

Supplementary Figure and Table

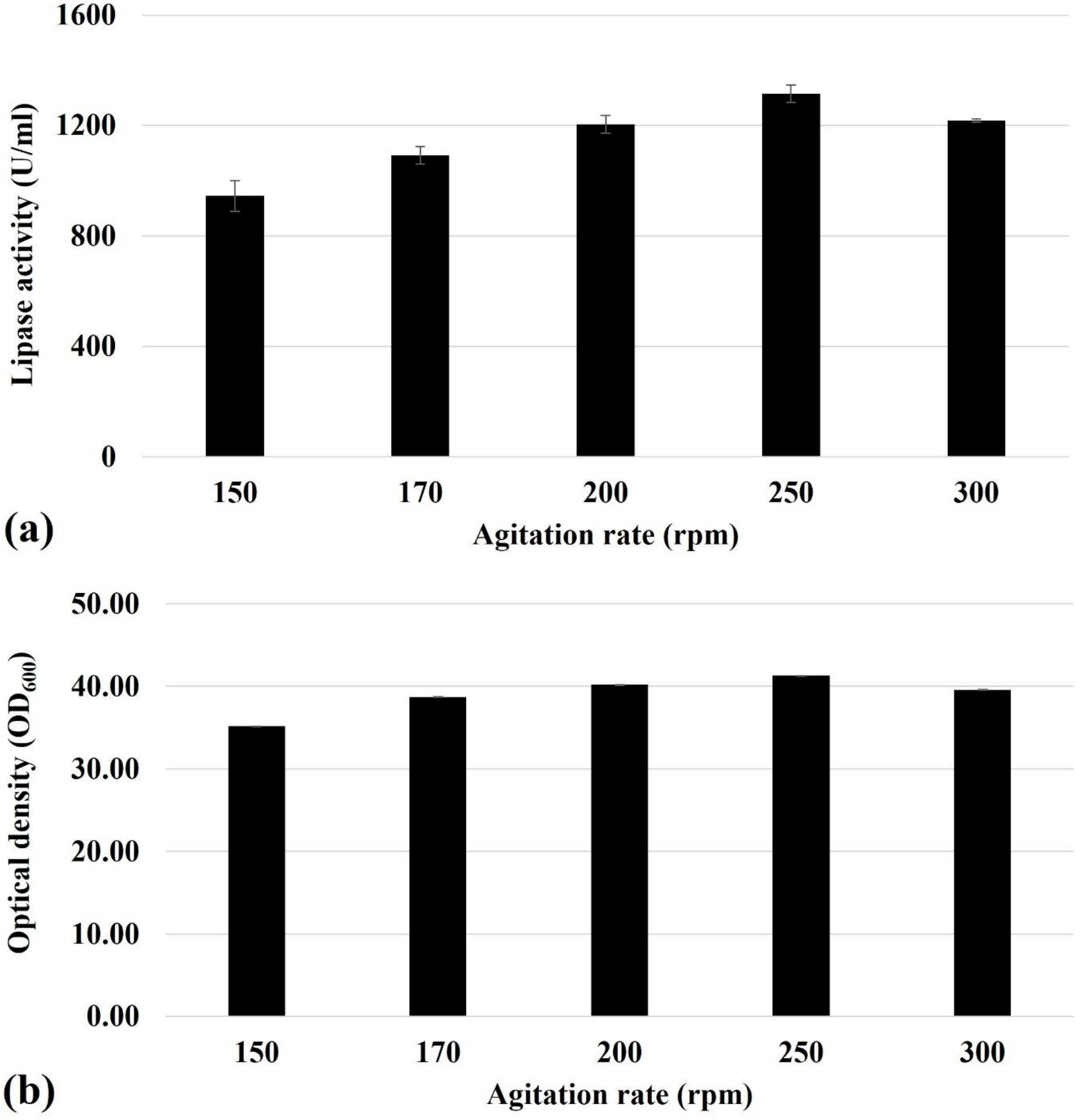

Figure. S1. Preliminary experiment in flask level to investigate minimum agitation rate to be set in fermenter level.

Table S1. Cost of each substrate and the calculated lipase activity cost of the corresponding substrate.

Substrate	Lipase activity (U/ml)	Cost of sugar (USD/g)	Cost per activity unit (USD/U)
Lactose	355.56	0.058	0.0016
Sucrose	251.85	0.020	0.0010
Sweet whey	288.89	0.001	0.0001

Note: The medium used in the experiment was supplemented with 1% (w/v) substrate. The prices of analytical reagent (AR) grade lactose and sucrose are 29.07 USD/500g and 12.46 USD/500g, respectively.

Table S2. Cost comparison of substrates used as medium constituents.

Medium components	Package (g)	Cost per package (USD)	Cost per gram (USD)
Lactose (Ajax)	500	29.07	0.058
Sweet whey	1000	1.48	0.001
Yeast extract (technical grade)	10000	563.56	0.056
Yeast extract (food grade)	10000	158.69	0.016
Olive oil	500	6.67	0.013
Palm oil	1000	1.42	0.001
